# Outcome of Initial Progression During Nivolumab Treatment for Hepatocellular Carcinoma: Should We Use iRECIST?

**DOI:** 10.3389/fmed.2021.771887

**Published:** 2021-12-13

**Authors:** Dong Ho Lee, Sangyoun Hwang, Young Hwan Koh, Kyung-Hun Lee, Ju Yeon Kim, Yoon Jun Kim, Jung-Hwan Yoon, Jeong-Hoon Lee, Joong-Won Park

**Affiliations:** ^1^Department of Radiology, Seoul National University Hospital, Seoul, South Korea; ^2^Institute of Radiation Medicine, Seoul National University Hospital, Seoul, South Korea; ^3^Department of Internal Medicine, Dongnam Institute of Radiological & Medical Sciences, Busan, South Korea; ^4^Center for Liver and Pancreato-Biliary Cancer, National Cancer Center, Goyang, South Korea; ^5^Department of Internal Medicine, Seoul National University College of Medicine, Seoul, South Korea; ^6^Liver Research Institute, Seoul National University College of Medicine, Seoul, South Korea

**Keywords:** liver cancer, iRECIST, pseudoprogression, progression, nivolumab

## Abstract

Immune response evaluation criteria in solid tumors (iRECIST) is recommended during immune checkpoint inhibitors (ICIs) treatment, due to the possibility of pseudoprogression. We evaluated the frequency of pseudoprogression in hepatocellular carcinoma (HCC) patients. This retrospective multicenter study involved 158 consecutive patients who underwent nivolumab treatment for HCC in Korea. At the initial evaluation, 94 patients presented with immune unconfirmed progressive disease, and 22 continued nivolumab. At the second evaluation, 21 of the 22 patients (95.5%) had confirmed progression and no pseudoprogression was observed. Considering low possibility of pseudoprogression, iRECIST may not be required for HCC.

## Introduction

Two immune checkpoint inhibitors (ICIs), nivolumab and pembrolizumab (programmed death-1 inhibitors), have been approved as second-line treatment options for advanced hepatocellular carcinoma (HCC) after failure of or intolerance to sorafenib (1–3). In addition, atezolizumab (a programmed death-ligand 1 inhibitor) and bevacizumab in combination showed superior overall survival as a first-line treatment for advanced HCC compared to sorafenib (4, 5). Therefore, ICIs are expected to be used more widely for HCC patients. However, there have been no validated biomarker for guide treatment (6). As ICIs can result in pseudoprogression in some types of tumors, which is an atypical response pattern defined by initial radiological progression followed by regression, immune response evaluation criteria in solid tumors (iRECIST) was created to accurately assess the treatment response (7). The unique feature of iRECIST is the assessment of the initial response after therapy: when progression is noted during the first response evaluation, it is classified as immune unconfirmed progressive disease (iUPD), indicating that further confirmation of progression is required. If disease progression is present after continued therapy for iUPD, the label of confirmed progression (iCPD) can be assigned. However, the clinical value of iRECIST in HCC patients treated with ICI has yet to be assessed. Therefore, we aimed to determine whether there is a pseudoprogression in HCC patients treated with ICIs.

## Methods

**Figure 1:**
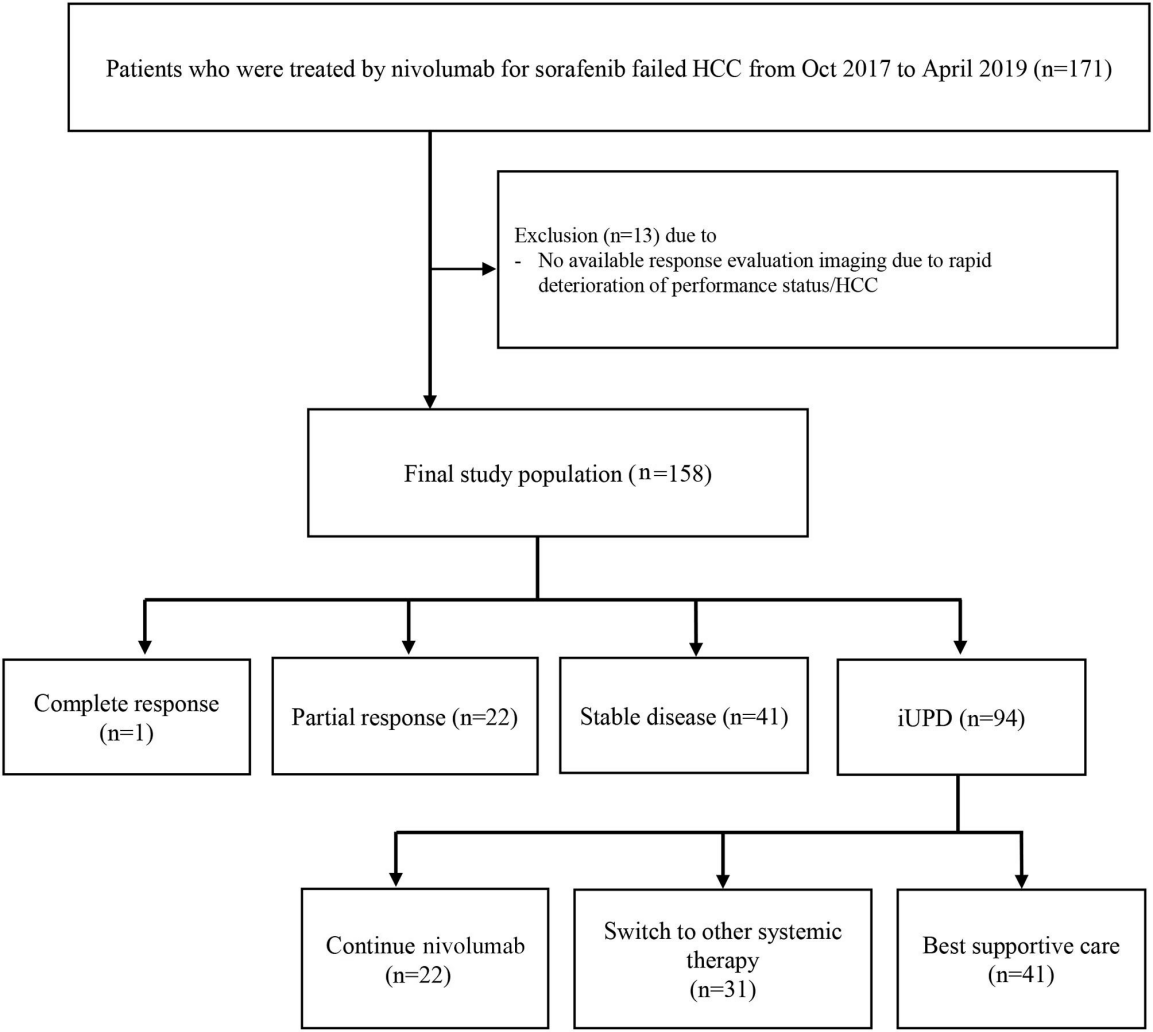
Patient enrollment process. iUPD, immune unconfirmed progressive disease.

This retrospective study involved 158 patients (130 males, median age = 62.0 years) who underwent nivolumab treatment for HCC after sorafenib failure between October 2017 and April 2019 at three referral hospitals in Korea: Seoul National University Hospital, National Cancer Center, and Dongnam Institute of Radiological & Medical Sciences (Figure 1). Patients who did not undergo response evaluation were excluded. HCC was diagnosed by either histopathology (*n* = 99, 62.7%) or imaging criteria (*n* = 59, 37.3%) according to the Korean Liver Cancer Association-National Cancer Center Korea Guidelines (8). Patients were treated by a standard dose of nivolumab (3 mg/kg intravenous infusion) every 2 weeks. Tumor assessment was performed using dynamic computed tomography (CT) or magnetic resonance imaging (MRI) after each 3–4 infusions. The response evaluation was carried out as outlined by iRECIST. All scans were reviewed by two independent radiologists at each site with >5 years of experience. If a patient continued treatment with nivolumab after iUPD, a second response evaluation was performed to identify iCPD or pseudoprogression. This study was approved by the institutional review board of each participating center. The requirement for written informed consent from patients was waived by the institutional review board because clinical data were analyzed anonymously in this study.

## Results

**Figure 2:**
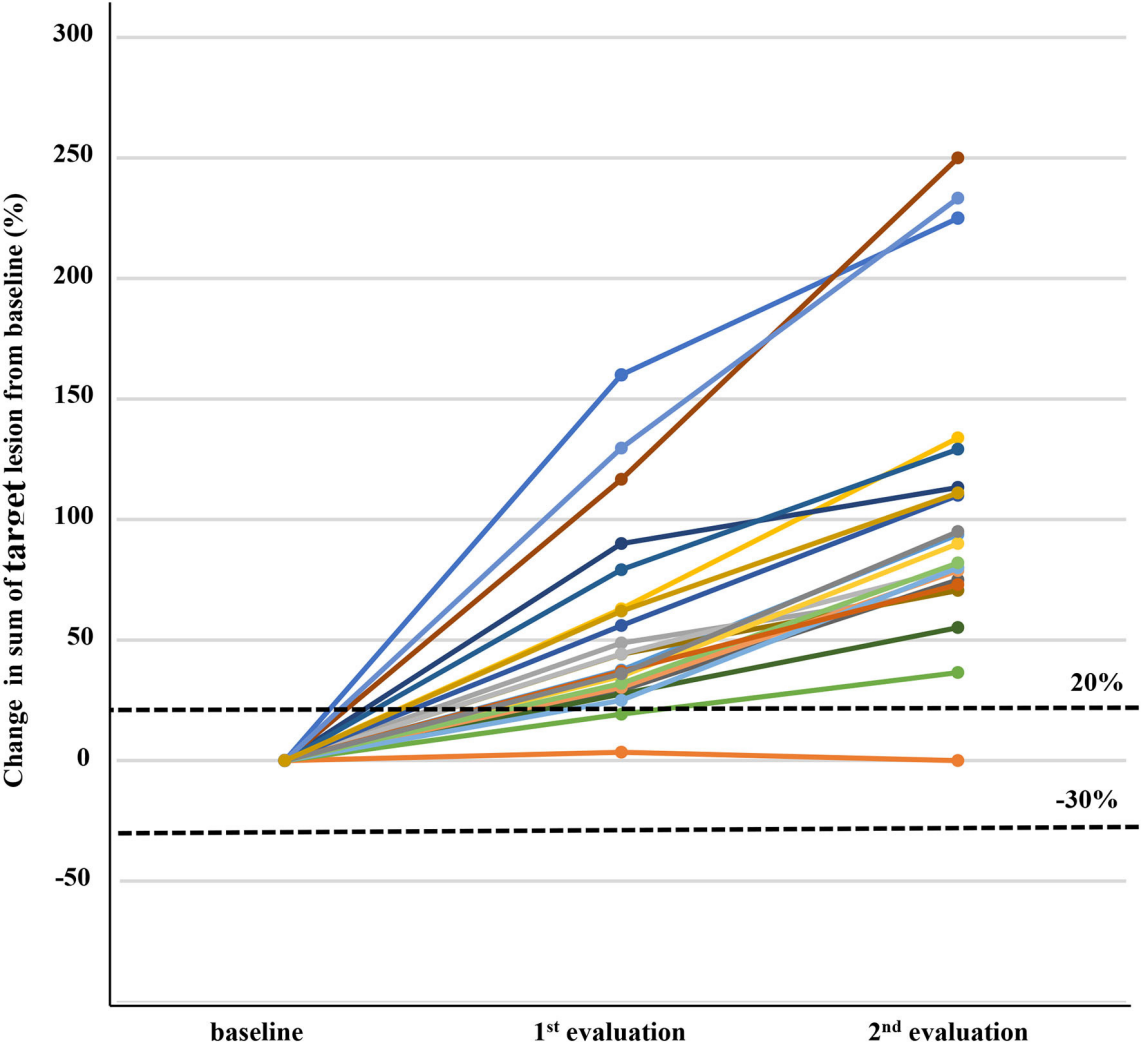
Changes in target lesions from baseline in 22 patients with immune unconfirmed progressive disease who continued immune checkpoint inhibitor treatment and subsequent response evaluation. Three patients in the first response evaluation and seven patients in the second response evaluation did not show more than 20% increase of sum of target lesions, but were assigned as progressive disease because of new lesion development.

At baseline, most patients had advanced HCC except one patient with intermediate stage HCC. The initial response to nivolumab was evaluated after median of 46 days (range = 14–94 days); complete response, partial response, stable disease, and iUPD was observed in 1, 22, 41, and 94 patients, respectively. The overall objective response rate was 14.6% (23/158) and the disease control rate was 40.5% (64/158). Among the 94 iUPD patients, 22 patients (23.4%) continued nivolumab treatment, 31 were switched to other systemic therapies, and 41 received best supportive care. Most of 72 patients who discontinued nivolumab after the first progression were accompanied by clinical progression of HCC with increasing serum alpha-fetoprotein level. For the 22 iUPD patients who continued nivolumab, a second response evaluation consisting of a follow-up dynamic imaging including CT and MRI (range = 20–119 days; median interval = 50 days) was conducted (Figure 1). At this second evaluation, iCPD was identified in 21 patients (95.5%) (Table 1). No one showed any regression of target lesions after iUPD (Figure 2). The one remaining patient with iUPD showed a stable tumor burden between the second response evaluation and first response evaluation, which indicated progressive disease compared to the baseline CT. This patient was subjected to iUPD again and planned to continue with nivolumab treatment. However, the patient experienced rapid deterioration of liver function, nivolumab therapy was stopped. Thus, the final response for this patient was iUPD. No pseudoprogression was observed. The initial response and reason for progression, as well as the treatment after iUPD and the second response to nivolumab continuation, are summarized in Table 1.

**Table 1:** Treatment response after immune checkpoint inhibitor with nivolumab for HCC patients.

	***N* (%)**
**Response (*****n*** **=** **158)**	
CR	1 (0.6%)
PR	22 (13.9%)
SD	41 (25.9%)
PD (iUPD)	94 (59.6%)
Progression of target lesion	17
Unequivocal progression of non-target lesion	3
Development of new lesion	14
Progression of both target and non-target lesion	22
Progression of target lesion + new lesion	16
Unequivocal progression of non-target lesion + new lesion	6
Progression of target, non-target lesion + new lesion	16
**Treatment after the first iUPD (*****n*** **=** **94)**	
Continue Nivolumab	22 (23.4%)
2nd response: iCPD	21 (95.5 %)
Progression of target lesion	7
Development of new lesion	3
Progression of non-target lesion + new lesion	7
Progression of target, non-target lesion + new lesion	4
2nd response: iUPD	1 (4.5%)
Switch to another systemic therapy	31 (33.0%)
Regorafenib	13
Cabozantinib	3
5-fluorouracil plus cisplatin	8
Lenvatinib	7
Stop active treatment except best supportive care	41 (43.6%)

*CR, complete response; PR, partial response; SD, stable disease; PD, progressive disease; iUPD, immune unfirmed progressive disease*.

## Discussion

Pseudoprogression is not an actual progression of tumor itself, but is a radiological increase in tumor size along with histological changes related to inflammatory responses (i.e., infiltration of immune-cells, such as cytotoxic T cells), edema, and necrosis. In addition, a delayed immunological response may be responsible for delayed tumor regression in patients after initial progression (9). Pseudoprogression during ICI therapy was first reported in melanoma patients treated with ipilimumab, an anti-cytotoxic T lymphocyte-associated antigen-4 monoclonal antibody (10). After the first report, variable rates of pseudoprogression, ranging from 2.8 to 11.1%, were reported for various tumors after ICI therapy (11). Given the possibility of pseudoprogression, which has rarely been reported with conventional cytotoxic chemotherapeutic agents and tyrosine kinase inhibitors (12), response evaluation during ICI therapy has been challenging. According to iRECIST, patients presenting with iUPD should receive continued treatment to confirm the final response. However, if the chance of pseudoprogression in HCC is sufficiently low, it may be more cost-effective and beneficial for the patient to switch to another therapy. In this study, we retrospectively evaluated treatment response during nivolumab in advanced HCC patients, focusing on the treatment method and outcome after initial iUPD. Among 22 patients who continued nivolumab after an initial iUPD, most patients (95.5%) showed iCPD at the second response evaluation and iUPD was assigned again in one patient. There were no cases of pseudoprogression observed in this study and thus, iRECIST was almost identical to RECIST. Considering these results, continuation of nivolumab for advanced HCC patients with iUPD may not be worthwhile, as the expected clinical benefit would be insignificant due to the low possibility of pseudoprogression. Similarly, another recent study recommended against the continuation of ICIs beyond progression in melanoma patients because of the unproven clinical benefit (13). However, current study has several limitations. This study was a retrospective one and the number of iUPD patients with continued ICI treatment in this study was small (22 patients). This study included only patients who were treated with nivolumab. Thus, further prospective studies with larger patient numbers who undergoing various ICIs combined with/without another ICI or multikinase inhibitors are warranted. In summary, almost all of the patients with iUPD in our study were confirmed progression upon subsequent evaluation. Therefore, RECIST rather than iRECIST may be appropriate for evaluating the tumor response in HCC patients undergoing ICI treatment, which would be a mainstay of systemic treatment for HCC in near future.

## Data Availability Statement

The original contributions presented in the study are included in the article/supplementary material, further inquiries can be directed to the corresponding author/s.

## Ethics Statement

The studies involving human participants were reviewed and approved by Institutional Review Boards of Seoul National University Hospital, National Cancer Center, and Dongnam Institute of Radiological & Medical Sciences. Written informed consent for participation was not required for this study in accordance with the national legislation and the institutional requirements.

## Author Contributions

DHL, J-HL, and J-WP: study concept, design, and drafting of the manuscript. DHL, SH, YHK, YJK, J-HY, J-HL, and J-WP: acquisition, analysis, or interpretation of data. J-HL and J-WP: had full access to all of the data in the study and takes responsibility for the integrity of the data and the accuracy of the data analysis. All authors: critical revision of the manuscript for important intellectual content and approved the final version of publication.

## Conflict of Interest

YJK reports consultant roles for Gilead, Bayer, Ono, and AbbVie; honoraria from Bayer and Gilead; and research funding from BTG, Bayer, Ono, Astra-Zeneca, Roche, LG, and BMS. J-HL reports honoraria from Gilead, Greencross Cell. J-WP reports consultant roles for BMS, Ono, Bayer, Eisai, Medatech, Roche, and Cue; and honoraria from Eisai, Bayer, and Ono. The remaining authors declare that the research was conducted in the absence of any commercial or financial relationships that could be construed as a potential conflict of interest.

## Publisher's Note

All claims expressed in this article are solely those of the authors and do not necessarily represent those of their affiliated organizations, or those of the publisher, the editors and the reviewers. Any product that may be evaluated in this article, or claim that may be made by its manufacturer, is not guaranteed or endorsed by the publisher.
